# Diagnostic Accuracy of Computer Tomography Angiography and Magnetic Resonance Angiography in the Stenosis Detection of Autologuous Hemodialysis Access: A Meta-Analysis

**DOI:** 10.1371/journal.pone.0078409

**Published:** 2013-10-23

**Authors:** Bin Li, Qiong Li, Cong Chen, Yu Guan, Shiyuan Liu

**Affiliations:** 1 Department of Radiology, Shanghai Changzheng Hospital, Second Military Medical University, Shanghai, China; 2 Radiation Treatment Center, 100 Hospital of PLA, Suzhou, Jiangsu Province, China; IRCSS - Istituto di Ricerche Farmacologiche Mario Negri, Italy

## Abstract

**Purpose:**

To compare the diagnostic performances of computer tomography angiography (CTA) and magnetic resonance angiography (MRA) for detection and assessment of stenosis in patients with autologuous hemodialysis access.

**Materials and Methods:**

Search of PubMed, MEDLINE, EMBASE and Cochrane Library database from January 1984 to May 2013 for studies comparing CTA or MRA with DSA or surgery for autologuous hemodialysis access. Eligible studies were in English language, aimed to detect more than 50% stenosis or occlusion of autologuous vascular access in hemodialysis patients with CTA and MRA technology and provided sufficient data about diagnosis performance. Methodological quality was assessed by the Quality Assessment of Diagnostic Studies (QUADAS) instrument. Sensitivities (SEN), specificities (SPE), positive likelihood ratio (PLR), negative likelihood values (NLR), diagnostic odds ratio (DOR) and areas under the receiver operator characteristic curve (AUC) were pooled statistically. Potential threshold effect, heterogeneity and publication bias was evaluated. The clinical utility of CTA and MRA in detection of stenosis was also investigated.

**Result:**

Sixteen eligible studies were included, with a total of 500 patients. Both CTA and MRA were accurate modality (sensitivity, 96.2% and 95.4%, respectively; specificity, 97.1 and 96.1%, respectively; DOR [diagnostic odds ratio], 393.69 and 211.47, respectively) for hemodialysis vascular access. No significant difference was detected between the diagnostic performance of CTA (AUC, 0.988) and MRA (AUC, 0.982). Meta-regression analyses and subgroup analyses revealed no statistical difference. The Deek’s funnel plots suggested a publication bias.

**Conclusion:**

Diagnostic performance of CTA and MRA for detecting stenosis of hemodialysis vascular access had no statistical difference. Both techniques may function as an alternative or an important complement to conventional digital subtraction angiography (DSA) and may be able to help guide medical management.

## Introduction

With increasing numbers of patients who suffered from end-stage renal disease and under long-term hemodialysis, the functioning vascular access related to better prognosis and quality of life is essential[1]. Nowadays the autologuous arteriovenous fistula (AVF) and the synthetic arteriovenous graft (AVG) remain the major access alternatives of choice [2], which have the advantage of long-term survival. However, problems including stenosis, thrombosis, failing to mature and so on might develop after the access creation. For prolonging life, stenosis, one of the major complications, leading to reduced blood flow and finally thrombosis even failure should be protected against especially. Therefore, early diagnosis of the presence, location and extent of the lesion and prompt salvage are imperative for the patency and function of the hemodialysis access [3]. 

Several imaging modality has been published in detection and depiction of the vascular access stenosis such as color Doppler ultrasonography (CDUS), computed tomography angiography (CTA), magnetic resonance angiography (MRA) and digital subtraction angiography (DSA) in recent years. Digital subtraction angiography (DSA) is a standard technique combined with the diagnosis and treatment for AVF or AVG dysfunctions currently [4]. However, there are several limitations such as exposure to radiation and the invasive procedure in clinical practice. The CDUS, an inexpensive and practical method, is readily available in AVF dysfunctions. But it is still operator dependent and limited for central venous assessment [5]. Although computed tomography angiography is rapid, effective, practical and non-invasive technique showing vascular anatomy and widely used in evaluating vascular tree in whole body, its ionizing radiation is difficult to overcome [4]. MRA which has been recently introduced for the evaluation of vascular access failure, is noninvasive, lacks ionizing radiation, but still limited for many flow-related artifact, claustrophobic patients, and limited field-of-view[6].

Considering that these controversial results, we performed this meta-analysis in an attempt to derive a more precise, comprehensive assessment for the overall diagnostic value of CTA and MRA in evaluation of vascular access in hemodialysis patients. To the best of our knowledge, this is the first meta-analysis on CTA and MRA in evaluation of vascular access in hemodialysis patients.

## Methods

### Publication search

Pubmed, MEDLINE, EMBASE, Cochrane Library database were all searched (Last search was updated on May, 2013). The following terms were used in searching: (vascular access or arteriovenous fistula or arteriovenous graft) and (hemodialysis or uremic or renal failure or renal disease or kidney failure or kidney disease) and (computed tomography angiography or magnetic resonance angiography or CT angiography or MR angiography or CTA or MRA). All the searched studies were retrieved, and their references were checked as well for other relevant publications. We also review articles to find additional eligible studies.

### Inclusion and Exclusion Criteria

Studies meeting the following selection criteria were included in this meta-analysis: (1) evaluation of the diagnostic performance of CTA or MRA for detecting or evaluating stenosis , (2) On per-segment or per-patient statistical basis, presentation of information for true-positive(TP), false-positive(FP), true-negative(TN) and false-negative(FN) results either found or calculated from data in the original published study, (3) Articles were published in English, (4) DSA or surgery should be the reference standards. Studies were excluded if not relevant to CTA or MRA for detecting or evaluating stenosis or without sufficient data obtained or duplicate publications. 

### Data Extraction and Quality Assessment

Relevant studies were examined by two independent observers (Bin Li and Qiong Li) with the Quality Assessment of Diagnostic Studies (QUADAS) [7] tool specifically developed for systematic reviews of diagnostic test accuracy. Data extraction including characteristics of the study population, methodological details for CTA, MRA, reference standard and outcome data was performed independently and discrepancies were resolved by discussion by 2 reviewers (Cong Chen and Yu Guan). The relevant data (TP, FP, TN, FN) were extracted into designed data collection forms. For optimal planning before salvage for dysfunction hemodialysis access, it is essential to have information on both the presence and extent of disease. Many studies subdivided the vascular access into multiple segments. A segment with more than 50% stenosis or an occlusion was considered diseased. A segment with 50% or less stenosis was considered nondiseased. 

### Meta Analysis

Diagnostic performance estimates for detecting more than 50% stenosis, such as sensitivity, specificity and likelihood ratio were calculated and pooled on a per-segment or per-patient basis. Using random-effects or fixed-effects model depends on the presence of statistical heterogeneity. Heterogeneity was explored by likelihood Chi-square Value (χ^2^) test and the inconsistency index (I^2^) [8]. P-value < 0.05 or I^2^ >50% suggested heterogeneity; A random effects model was for the meta-analysis to obtain a summary accuracy parameter if heterogeneity was identified; otherwise a fixed effects model was used.

One of main causes of heterogeneity is threshold effect in test accuracy studies. The threshold effect arises owing to different thresholds or cut-offs used in different studies to define a positive (or negative) test result [9]. If threshold effect exists, which was assessed by computating Spearman correlation between the logit of sensitivity and logit of (1-specificity), there is a positive correlation between sensitivities and 1-specificities (or a negative correlation between sensitivities and specificities). A positive correlation (P < 0.05) suggested the threshold effect. If heterogeneity due to threshold effect was present, the accuracy data should be pooled by fitting a SROC curve and calculating the area under the curve (AUC).

Apart from the threshold effect, in test accuracy studies, several other factors can contribute to heterogeneity. If there was no threshold effect but significant heterogeneity, a regression meta-analysis and subgroup analysis was performed because assessment should only be attempted within homogeneous subgroup.

Publication biases were assessed by Deeks's funnel plots.

All the statistical computations were performed using the Meta-Disc software version 1.4 [9] and the Stata/SE statistical software version 12.1 (StataCorp LP, Texas, USA). P values of less than 0.05 were considered to be statistically significant.

## Results

### Eligible studies

**Figure 1:**
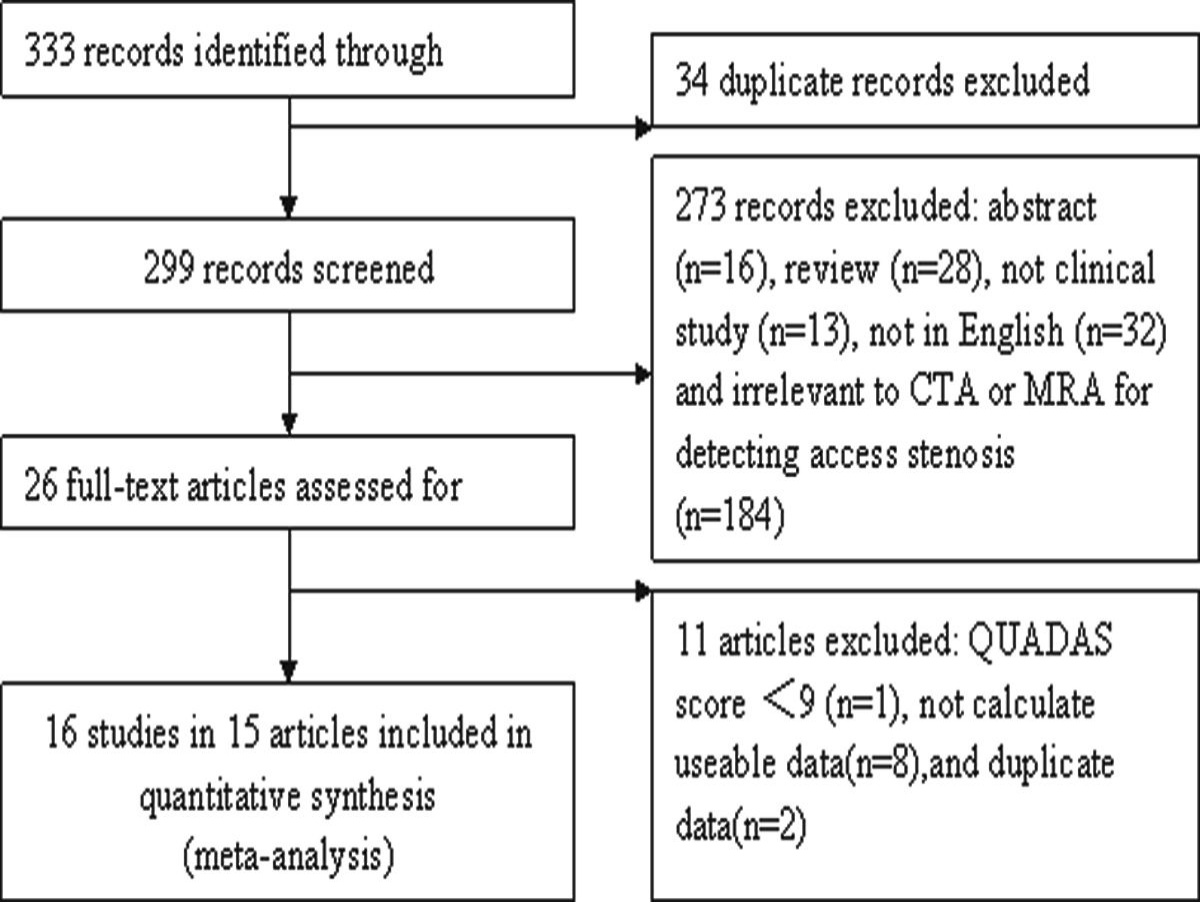
Flowchart of study identification, inclusion, and exclusion.

Our search strategy identified 333 primary studies. After ruling out the obviously irrelevant abstracts, 26 studies were left and their full texts were obtained. Figure **1** outlines our study selection process. The search initially yielded 333 primary studies. Finally there were 16 studies in 15 articles included in the meta-analysis [10-24]. The main reasons for exclusion were as follows: (1) not relevant to CTA or MRA for detecting or evaluating stenosis; (2) unable to create 2×2 table; (3) QUADAS score less than 9. The characteristics of the each study included are presented in Table **1**.

**Table 1:** Studies included in the meta-analysis.

Author	Year		Patient Num	CT Slices / MR Strength Field	Access Type	Calculating Basis	Contrast Enhancement	Noninvasive Modalities	TP	FP	FN	TN
Cansu[10]	2013	41		64	AVF and AVG	segment	yes	CTA	34	2	1	30
Wasinrat[24]	2011	21		64	AVF	segment	yes	CTA	32	6	0	109
Rooijens[21]	2008	15		4	AVF and AVG	segment	yes	CTA	9	1	2	124
Heye[16]	2009	36		64	AVF	segment	yes	CTA	46	8	5	103
Dimopoulou[12]	2011	24		16	AVF and AVG	segment	yes	CTA	37	0	2	33
Ko[17]	2005	36		4	AVF and AVG	segment	yes	CTA	126	2	2	69
Lin[19]	1998	9		4	AVF	patient	yes	CTA	6	0	0	3
Cavagna[11]	2000	13		4	AVF	patient	yes	CTA	11	0	0	2
Froger[15]	2005	48		1.5	AVF and AVG	segment	yes	MRA	68	3	2	209
Waldman[23]	1996	13		0.5	AVF and AVG	segment	no	MRA	8	1	0	33
Takahashi[22]	2004	15		1	AVF	segment	yes	MRA	16	3	3	19
Duijm[14]	2006	101		1.5	AVF and AVG	segment	yes	MRA	18	1	0	82
Doelman[13]	2005	81		1.5	AVF and AVG	segment	yes	MRA	106	7	5	315
Planken[20]	2003	15		1.5	AVF and AVG	patient	yes	MRA	10	4	0	1
Cavagna[11]	2000	13		0.5	AVF	patient	yes	MRA	10	0	1	2
Laissy[18]	1999	19		1	AVF and AVG	patient	no	MRA	11	1	1	6

Calculating Basis means stenosis number count by lesion per patient or lesion per vascular access segment.

TP=true positive; FP=false positive; FN=false negative; TN=true negative

The details about the data acquisitions of CTA and MRA are summarized in Table **S1**.

### Threshold effect analyze

Spearman correlation coefficient was determined to be 0.071 (P=0.867) and -0.677 (P=0.071) for CTA and MRA respectively, which indicated absence of threshold effect that could cause variations in accuracy estimates among the individual studies. 

### Data synthesis

**Figure 2:**
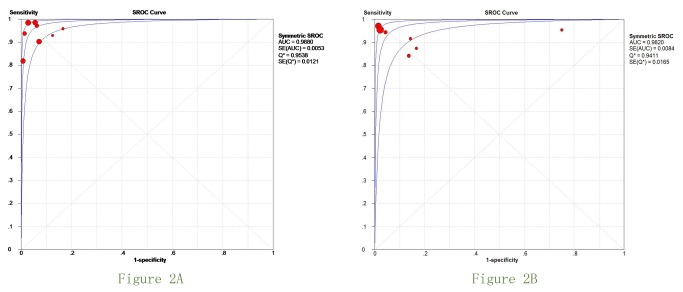
Summary ROC (SROC) curves for CTA and MRA.

Figure **2 (A-B)** shows the SROC curves of the performance of CTA and MRA. Overall AUC of CTA and MRA was 0.988 and 0.982, which suggesting good diagnostic accuracy. Pair-wise comparisons confirmed no statistical difference between CT and MR imaging performance. For each technique, the weighted summary of sensitivity, specificity, positive likelihood ratio, negative likelihood ratio, DOR, P value for heterogeneity, and I^2^ value are summarized in Table **2**. No 95%CIs of OR included 1, confirming the diagnostic value of all modalities. 

**Table 2:** Weighted summary of sensitivity, specificity, and OR for each modality.

Modality		Sensitivity	Specificity	PLR	NLR	DOR		AUC	Threshold Effect
CTA								AUC=0.9880	P=0.867
	Pooled estimates	0.962	0.961	17.64	0.06	393.69			
	95%CI	0.93-0.98	0.94-0.98	11.17-27.84	0.03-0.12	155.20-998.67			
	P value*	P=0.068	P=0.119	P=0.369	P=0.179	P=0.287			
	I2 value	46.90%	39.00%	7.90%	31.20%	18%			
MRA								AUC=0.982	P=0.071
	Pooled estimates	0.954	0.971	13.36	0.075	211.47			
	95%CI	0.920-0.976	0.955-0.982	2.42-73.95	0.039-0.144	46.36-964.67			
	P value*	P=0.327	P=0.000	P=0.000	P=0.203	P=0.001			
	I2 value	13.20%	79.30%	95.20%	28.20%	70.40%			

**Figure 3:**
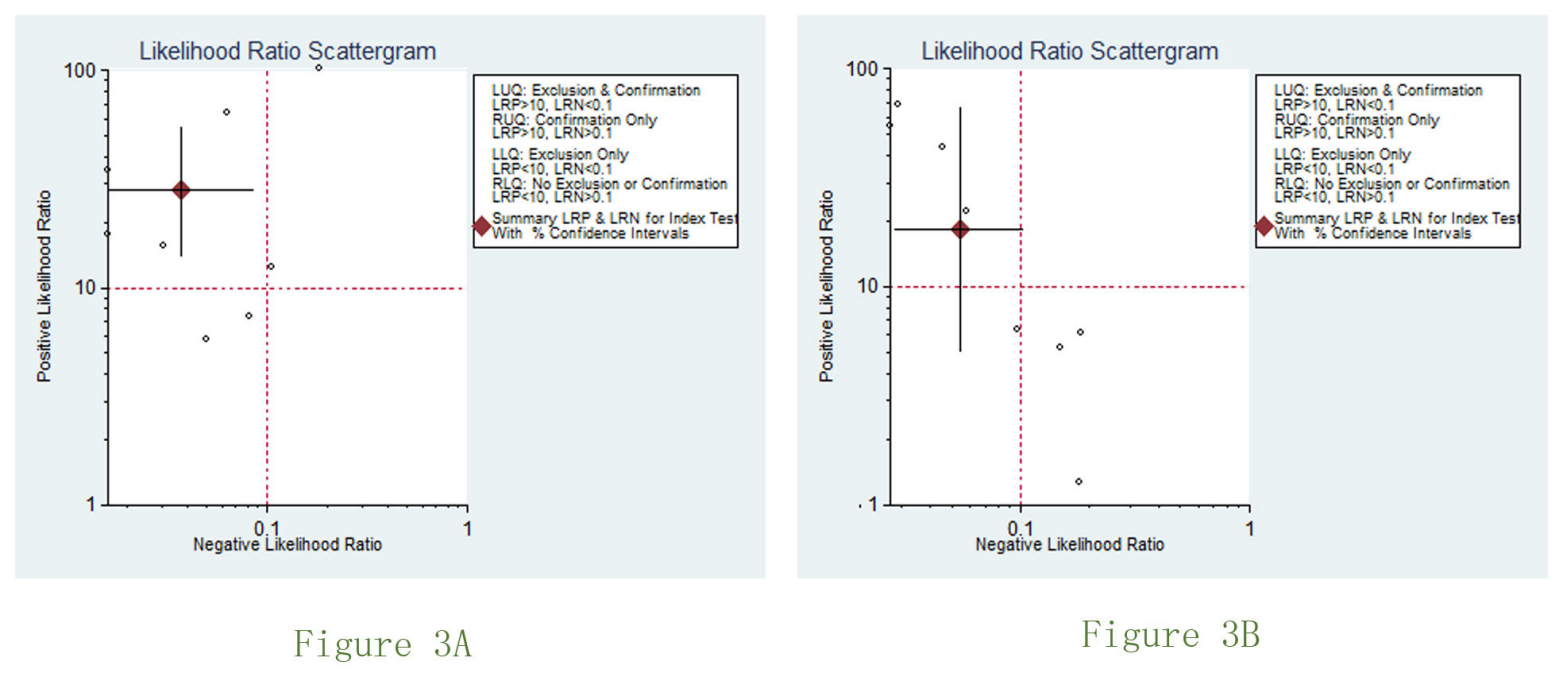
Scattergram of the positive likelihood ratio and negative likelihood ratio.

There was substantial between-study heterogeneity (P < 0.05 and I2 > 50%) for specificity, PLR and DOR in MRA imaging studies. To explore possible explanations for the heterogeneity, we firstly applied meta-regression analysis by adding the number of patients, year of publication, MR field strength, CT slice thickness, whether using contrast enhancement, access type, calculating stenosis number by patient or vascular access segment as variates. No apparent relationships were found (P > 0.05).Subgroup analyses were then conducted based on the type of access (AVF and AVF versus AVF), MR field (>1T versus ≤1T), lesion calculating( numbers per patient versus numbers per access segment) for MRA studies. Still, no significant difference was found (P > 0.05). The results of Deeks’ funnel plot asymmetry test (P =0.035) showed strong evidence for publication bias for MRA studies but no publication bias was indicated for CTA studies. Scattergram of the positive likelihood ratio and negative likelihood ratio is shown in Figure **3 (A-B)**. 

## Discussion

Hemodialysis vascular access is considered to be critically important for patients with end-stage renal disease, especially arteriovenous grafts (AVGs) and arteriovenous fistulas (AVFs) mainly including the brachiocephalic, brachiobasilic and radiocephalic arteriovenous fistula [1]. The autologuous hemodialysis access, which had been initially proposed since 1966, is common autologous vascular access for hemodialysis [25]. However, dysfunction of the vascular access remains a common and costly problem in patients who are dependent on hemodialysis for survival [26]. The common causes for access dysfunction or failure include stenosis, thrombosis, infection, and aneurysm formation. Among these, stenosis is probably the most important. Although vascular stenosis most commonly occur at the arteriovenous anastomosis of an AVF or at the venous anastomosis of an AVG, but can also occur distant from the access site [27]. In such situations, diagnostic studies to evaluate the patency of the entire central arterial or venous system and detect stenosis become important. Imaging is necessary for evaluating complication and planning salvage measure in patients with dysfunction autologuous hemodialysis access. Noninvasive imaging modalities, including duplex ultrasonography, computed tomography angiography (CTA) and magnetic resonance angiography (MRA) are available for detecting and grading stenosis. 

CTA and MRA can also diagnose vascular impairment other than stenosis, such as aneurysm, thrombosis and pseudoaneurysms, provides excellent preoperative and postoperative evaluation of patients with AVF malfunction and generated a “road map” for therapy planning [28-30]. 

Both CTA and MRA for autologuous hemodialysis access have some advantages and disadvantages.

Apart from two studies [18,23] about MRA that didn’t use any contrast agents, all other studies applied gadolinium contrast agent for MRA and nonionic iodinated agent for CTA. In terms of the use of contrast agents, the gadolinium contrast agent for MRA does not outweigh the iodinated agent for CTA because both contrast material have side effects. The iodinated agent may cause allergic reactions. It even can induce a further deterioration of residual renal function in patients with renal insufficiency. Compared with the iodinated contrast, the gadolinium contrast agent has a relatively favorable safety profile. For the gadolinium contrast agent, although anaphylactic reactions are rare and the nephro-toxicity in impaired kidneys is low, late complication of nephrogenic systemic fibrosis (NSF) in using gadolinium agent may be associated with patients with renal insufficiency [31]. However, NSF must always be balanced versus the outcome of an investigation, respectively versus the outcome of a denied MRA.

NSF, which is regarded initially as possible complication of MRA, is a systemic disorder. It is an illness described in patients with kidney disease who present with firm, erythematous, and indurated plaques of the skin associated with subcutaneous edema. Primarily it involves the extremities and may result in flexion contractures with limited range of motion, pain, paresthesias, and/or severe pruritus. It may also involve other organs, including the lungs, heart, diaphragm, liver, and kidneys, resulting in variable end organ damage and even death [31]. The risk of NSF with MRA raises concerns over the safety of CE-MRA applications. The relationship between gadolinium-based contrast agents and NSF was firstly suggested in January 2006, when Grobner reported five patients who received a gadolinium-based contrast agent prior to the diagnosis of NSF [32]. Then more publications reported the link between high dose gadolinium agents and NSF in patients with renal insufficiency [31,33-35]. Most current theories implicate free Gd ions as a likely pathway for development of NSF and ultra-stable Gd formulations have theoretical advantages in reducing the risk of NSF. It is indicated that the risk of NSF with Gd use is dose dependent and may be related to the residence time of gadolinium within the circulation [31]. However, the precise relationship between gadolinium dose and risk of NSF remains unknown. Therefore, it is rational to use the minimum effective dose for CE-MRA in patients considered at elevated risk [36,37]. In studies included in our meta-analysis, CTA or MRA examination was scheduled a day before hemodialysis or patients underwent hemodialysis immediately after CTA or MRA examination, which might help to prevent the development of NSF.

There are contraindications and side effects for both CTA and MRA. The pregnant, children and patients with renal insufficiency should be suggested to avoid CTA examine because CTA examine exposes the patient to ionizing radiation and iodinated contrast agent [24]. The presence of claustrophobia, pacemaker, and magnetized metal in patients are limited in MR scanner. Gadolinium contrast agents are also nephrotoxic in patients with end-stage renal failure, even though the incidence is not as high as that of iodinated contrast agents [38]. Moreover, there are the possible link between gadolinium-based contrast agents and NSF.

From a cost standpoint, it might be worthwhile to consider performing CTA or MRA before DSA in all patients with failing hemodialysis access fistulas and grafts. Doelman [13] revealed that CTA and MRA costs were less than DSA ($ 200 vs. $283 vs. $375) in Netherlands. Whether these cost reductions and quality-of-life improvements outweigh the costs and burden of an extra work-up (if lesions are not be detected or diagnosed) should, of course, be addressed in a formal cost-effectiveness study. Visser [39] reported that the costs of contrast enhanced CT angiography, gadolinium enhanced MR angiography and DSA estimates $237, $574 and $1,183, respectively. They also performed a cost-effectiveness study and considered CTA has the potential to be more cost-effective compared with MRA.

As far as contrastload is concerned, it might be recommend considering MRA. Even at the higher dose of 0.3 mmol/kg, the MR contrast volume is still less than half that of the iodinated contrast required for CTA. 

Radiation dose might limit the practical application of CTA for the evaluation of dysfunctional hemodialysis fistulas, since multiple CTA could be effective for surveillance of patency rates but multiple CTA would expose the patient to additional radiation. Data about patient radiation dose are available in only one study [24] included in our meta-analysis. Wasinrat et al [24] recorded the volume CT dose index (CTDIvol), doselength product (DLP), and length of the scan for each patient. They calculated the length of each region and the effective doses by using the conversion factor of 0.012 mSv/ mGy.cm, 0.0023 mSv/mGy.cm, 0.0054 mSv/ mGy.cm, and 0.0017 mSv/ mGy.cm for the arm, head, neck, and upper chest, respectively. Results about radiation dose they obtained are as followings: the average CTDIvol of MDCT angiography was 14.39 ±2.02 mGy, the average DLP was 1163.49 ±174.52 mGy.cm and the calculated effective dose indices were 4.65 ±0.78 mSv. The radiation exposed to the upper chest region was 52% (2.4mSv in average) and only 1.28 mSv (25%) in average in the head and neck regions. They reported that although the scan length of the arm was quite long about 30 to 60 cm in CT acquisition, the conversion factor was little thus radiation exposure was minimal (1.06 mSv in average). For the reason, increasing the scan length to the distal forearm and hand did not significantly increase the overall radiation exposure. Although no information about patient radiation dose was available, Dimopoulou [12] suggested that since starting using MDCTA, the mean examination time and the frequency should be decreased. Further investigations of patient radiation dose are needed to obtain radiation dose of MDCT angiography and DSA.

Our meta- analysis, including data from 500 patients with autologuous hemodialysis access, showed that CTA and MRA were accurate modalities for detecting stenosis and both had similar diagnostic performance, which was in agreement with most previous literature that suggested MDCT and MR angiography as alternatives diagnostic techniques to DSA in the analysis of hemodialysis fistula and graft stenosis. Both CTA and MRA had high sensitivity, specificity, positive likelihood ratio, negative likelihood ratio and diagnostic OR. There was no significant difference between the performance of CTA and MRA for stenosis detecting.

Homogeneity test indicated that there’s no significant heterogeneity for CTA studies included but substantial heterogeneity existed in MRA studies for specificity, PLR and DOR. Meta-regression analyses showed that number of patients, year of publication, MR field strength, CT slice thickness, access type, calculating stenosis number by patient or vascular access segment had no significant influence on the between- study heterogeneity. Subgroup analyses were performed based on the type of access (AVF and AVF versus AVF), MR field (>1T versus ≤1T), lesion calculating( numbers per patient versus numbers per access segment) for MRA studies, No statistical difference was detected as well. Deek’s Funnel plots with marked asymmetry suggested a publication bias for MRA.

Therefore, conclusions of published studies in detecting and evaluating hemodialysis access stenosis using MRA may be overestimated, as studies with positive and favorable results are more likely to be accepted and published.

However, we could not ascribe all the heterogeneity to publication bias. Because significant disease may be detected by MRA and CTA and which allow 3-dimensional assessment but be unrecognized by the 2-dimensional DSA, it may lead to so called false-positive results from a professional and clinical standpoint. Furthermore, in 8 included studies, Planken et al reported a considerably low specificity (SPE=20%) for detection of significant stenosis, which had significant difference with others. We thought this study might have relationship with the above reason and result in between-study heterogeneity of our meta-analysis. Their large number of false-positive lesions detected with contrast- enhanced MR angiography also may be caused by a limited spatial resolution, which resulted in stenosis overestimation. Moreover, because of the use of a rectangular surface coil, no information could be obtained about the venous outflow of the upper arm.

One limitation of our study is that we could not present the exact reasons for heterogeneity which was observed for pooled specificity, PLR, DOR of MRA. Except for those discussed above, there are still many variables which differed among studies regarding patient position during examination, patient characteristics, acquisition protocol, image analysis technique, indication for imaging, interobserver variability, and quality of studies, especially patient position. So meta-regression analyses we performed to detect heterogeneity were still insufficient. But these factors were not taken into account and the effect of these variables could not be examined because of variation in data presentation or incomplete reporting of data. Besides that, the number of studies on non-contrast enhancement MRA included in this meta-analysis was not enough to enable us perform a reliable subgroup analysis. It was thought that Gd-enhanced MRA is more likely to avoid stenosis overestimation than non-contrast enhancement MRA. Unlike conventional MRA techniques (TOF and phase contrast), which rely on velocity-dependent inflow or phase-shift effects, employing gadolinium does not depend upon blood motion. Unfortunately, the effect cannot be explored. More studies should emphasize on non-contrast enhancement MRA evaluating hemodialysis access in the future. 

The second limitation is that there are differences of the field of view (FOV) between contrast enhancement MRA and non-contrast enhancement MRA. The FOV of contrast enhancement MRA is similar to that of CTA, which included the complete vascular tree comprising feeding artery, anastomosis, draining vein and central venous outflow up to the level of the superior vena cava. But the FOV of non-contrast enhancement MRA could not include the central venous outflow. Depiction of the central venous outflow is important because subclavian vein stenoses are frequently present in dialysis patients.

The third limitation stemmed from the fact that, for practical reasons, we included only studies that were written in English. Although certain less-qualified studies would be neglected by limiting publication language to English, it might invoke the so-called Tower of Babel bias [40]. The funnel plot for publication bias was indeed statistically significant. 

## Conclusions

In conclusion, despite the limitations of the present meta-analysis, results available indicate that CTA and MRA had similar, excellent accuracy for detecting stenosis of hemodialysis vascular access. There is no statistical difference between the diagnostic performance of CTA and MRA. Both techniques may function as an alternative or an important complement to conventional digital subtraction angiography and may be able to help guide medical management.

## Supporting Information

Table S1 **Summary of details about the data acquisitions of CTA and MRA.**
(DOC)

Checklist S1 **PRISMA Checklist.**
(DOC)
