# Supplementary material for: Diagnostic Accuracy of Computer Tomography Angiography and Magnetic Resonance Angiography in the Stenosis Detection of Autologuous Hemodialysis Access: A Meta-Analysis
Source: PLoS One. 2013 Oct 23;8(10):e78409. doi: 10.1371/journal.pone.0078409 (PMC3806799; doi:10.1371/journal.pone.0078409)
Supplement: Table S1 — Summary of details about the data acquisitions of CTA and MRA. (DOC) [file pone.0078409.s001.doc]

**Table S1** Summary of details about the data acquisitions of CTA and MRA

| Author | Year | CE | Modalities | Contrast Material | Scan Protocol | Post processing | Patient Position |
| --- | --- | --- | --- | --- | --- | --- | --- |
| Cansu | 2013 | yes | CTA | lopromide,  300 mgI/mL,  4–6 mL/min | a pitch of 2; 0.5 s scanner rotation; 120 kV, 160 mAs; 38 cm field of view and 1 mm slice thickness;Bolus tracking:peak attenuation of the ascending aorta with a threshold level of 150 HU | MPR,MIP,VR | ND |
| Wasinrat | 2011 | yes | CTA | lopromide,  370 mgI/mL,  100mL bolus | 1.25 mm collimation;500 ms rotation time;120 kV and 200-220 mAs;from the mid level of the chest to the hand;Bolus tracking:peak attenuation of the ascending aorta with a threshold level of 150 HU | MPR,MIP,VR | supine position with raising arms over the head pointed to the gantry |
| Rooijens | 2008 | yes | CTA | lopromide,  3 mL/sec  100ml preloading | 2.5 mm collimation;from axilla to wrist to cover the arteriovenous anastomosis,the inflow artery and the outflow vein;Bolus tracking:selecting an engorged vessel proximal to the AVF with a threshold level of 120 HU | MPR,MIP,VR | ND |
| Heye | 2009 | yes | CTA | iomeprol,  2.2 mL/s:  100 mL | a pitch of 1.2,acquisition of 64 × 0.6 mm, rotation time of 0.5 second, 120 kV, and 180 mAs | MPR,MIP,VR | position on their side ipsilateral to the AVF with their arm extended above the head |
| Dimopoulou | 2011 | yes | CTA | lopromide,  370 mgI/mL,  100mL bolus | 120 kV, 140 mAs, 0.5 s gantry rotation speed, 0.75 mm collimation, 10.5 feed/rotation (pitch equivalent of 1.15) | MPR,MIP,VR | prone position with the AVF/AVG arm extended above the head. |
| Ko | 2005 | yes | CTA | Omnipaque  [iohexol],  370 mgI/mL,  100mL bolus,  2.5 mL/s | a pitch of 3, 0.5-sec scanner rotation, 120 kV, 160 mAs, and a 35-cm field of view.Bolus tracking:selecting an engorged vessel proximal to the AVF with a threshold level of 120 HU | MPR,MIP,VR | supine or prone position with the arm extended above the head or placed alongside the body |
| Lin | 1998 | yes | CTA | Optiray,  320mg/ml,  4ml/s,preload | multiple | MPR,MIP,SSD | ND |
| Cavagna | 2000 | yes | CTA | 100 ml,  3ml/s | 0.8-sec gantry rotation speed, 3-mm collimation, 5-mm/sec table speed (pitch 1.7) | MIP,SSD | lying with the arm extended |
| Froger | 2005 | yes | MRA | Prohance,  0.6 mL /kg  0.6 mL/sec | repetition time msec/echo time msec, 4.1/1.34; flip angle, 20°; acquisition matrix, 432 346; number of sections, 120; overcontiguous section thickness, 0.55 mm; acquisition voxel size, 1.00 1.00 1.10 mm | MPR,MIP | semioblique supine position on the side of the shunt arm with the upper extremities lying next to the body |
| Waldman | 1996 | no | MRA | -- | MPTFE sequence:the repetition time 20 ms, echo time 6.9 ms, the flip angle 50”. Two signal averages were obtained using a 240 X 240 mm2 field-of-view and a 128 x 256 acquisition matrix. PC MRA: Repetition time 17 ms, echo time 8.3 ms, the flip angle 20”. Velocity encoding 200 cm/s. | MPR,MIP | in the supine position |
| Takahashi | 2004 | yes | MRA | Omniscan,  10ml,  2 ml/s | TR/TE=6.1–6.2/1.4 ms, TI=23–30 ms, flip angle=30°,field of view (FOV) in the ratio of 5 to 3=24×14.4-30×18.0 cm, excitations=1-2,matrix=256×160 (pixel size=0.94×0.90-1.17×1.13), receiver bandwidth=31.2kHz, and acquisition time of single phase=21–37 s | MPR,MIP | ND |
| Duijm | 2006 | yes | MRA | Prohance;  20 mL,  0.6 mL/s | repetition time/echo time/flip angle,4.1/1.34/20; acquisition voxel size, 1.00×1.00×1.10 mm; interpolated voxel size, 0.84×0.84× 0.55 mm; acquisition matrix, 432×346 pixels; 120 slices; overlapping section thickness, 0.55 mm; imaging time, 32 seconds | MPR,MIP | in a semioblique supine position |
| Doelman | 2005 | yes | MRA | Prohance;  20 mL,  0.7 mL/s | repetition time/echo time/flip angle, 4.1/1.34/20;acquisition voxel size, 1.00×1.00×1.10 mm; interpolatedvoxel size, 0.84×0.84×0.55 mm; acquisition matrix, 432 346 pixels; 120 slices | MPR,MIP | in the supine position |
| Planken | 2003 | yes | MRA | Magnevist;  35 mL,  3ml/s | TR/TE＝5.1/1.5 msec;flip angle＝40°, FOV=300＝90 mm, matrix=304＝58.The acquisition voxel size was 3.1mm. | MPR,MIP | in the supine position |
| Cavagna | 2000 | yes | MRA | Omniscan,  0.1 mmol/kg,  2 ml/sec | coronal T1 FFE sequence: TR/TE 8.7/3.3, flip angle 60°,coronal plane, 1 NSA, FOV 450 mm, RFOV 55 mm, matrix 256×512, 25 slices (thickness 2.2 mm), acquisition time 13 sec | MPR | lying with the arm extended |
| Laissy | 1999 | no | MRA | -- | repetition time/echo time of 23/10 milliseconds, at a 60-degree flip angle. For each series, 40 to 50 successive sections were acquired using a field of view of 200 mm, a 128 × 256 acquisition matrix | MPR,MIP | in the supine position |

CE: Contrast Enhancement --: no use of contrast material MPR:: multiplanar reformation MIP: maximum intensity projection SSD:surface Shaded displa

| Laissy | 1999 | no | MRA | -- | repetition time/echo time of 23/10 milliseconds, at a 60-degree flip angle. For each series, 40 to 50 successive sections were acquired using a field of view of 200 mm, a 128 × 256 acquisition matrix | MPR,MIP | in the supine position |
| --- | --- | --- | --- | --- | --- | --- | --- |

CE: Contrast Enhancement --: no use of contrast material MPR:: multiplanar reformation MIP: maximum intensity projection SSD:surface Shaded displa
