# Supplementary material for: Diagnostic Accuracy of Computer Tomography Angiography and Magnetic Resonance Angiography in the Stenosis Detection of Autologuous Hemodialysis Access: A Meta-Analysis
Source: PLoS One. 2013 Oct 23;8(10):e78409. doi: 10.1371/journal.pone.0078409 (PMC3806799; doi:10.1371/journal.pone.0078409)
Supplement: Checklist S1 — PRISMA Checklist. (DOC) [file pone.0078409.s002.doc]

| **Section/topic** | **#** | **Checklist item** | **Reported on page #** |
| --- | --- | --- | --- |
| **TITLE** | | |  |
| Title | 1 | Diagnostic Accuracy of Computer Tomography Angiography and Magnetic Resonance Angiography in the Stenosis Detection of Native Hemodialysis Access: A Meta-Analysis | Title |
| **ABSTRACT** | | |  |
| Structured summary | 2 | Context: The diagnostic performances of computer tomography angiography (CTA) and magnetic resonance angiography (MRA) for detection and assessment of stenosis in patients with native hemodialysis access.  Objective: To compare the diagnostic performances of computer tomography angiography (CTA) and magnetic resonance angiography (MRA) for detection and assessment of stenosis in patients with native hemodialysis access.  Data Sources: Pubmed, MEDLINE, EMBASE and Cochrane Library database from January 1984 to May 2013.  Study Selection: Eligible studies were in English language, aimed to detect more than 50% stenosis or occlusion of native vascular access in hemodialysis patients with CTA and MRA technology and provided sufficient data about diagnosis performance.  Data Extraction: Independent extraction of articles by 2 authors using predefined data fields.  Data Synthesis: Sixteen eligible studies were included, with a total of 500 patients. Both CTA and MRA were accurate modality (sensitivity, 96.2% and 95.4%, respectively; specificity, 97.1 and 96.1%, respectively; DOR [diagnostic odds ratio], 393.69 and 211.47, respectively) for hemodialysis vascular access. No significant difference was detected between the diagnostic performance of CTA (AUC, 0.988) and MRA (AUC, 0.982). Meta-regression analyses and subgroup analyses revealed no statistical difference. The Deek’s funnel plots suggested a publication bias.  Conclusions: Diagnostic performance of CTA and MRA for detecting stenosis of hemodialysis vascular access had no statistical difference. Both techniques may function as an alternative or an important complement to conventional digital subtraction angiography (DSA) and may be able to help guide medical management. | Abstract |
| **INTRODUCTION** | | |  |
| Rationale | 3 | With increasing numbers of patients who suffered from end-stage renal disease and under long-term hemodialysis, the functioning vascular access related to better prognosis and quality of life is essential. early diagnosis of the presence, location and extent of the lesion and prompt salvage are imperative for the patency and function of the hemodialysis access. Several imaging modality has been published in detection and depiction of the vascular access stenosis. | Introduction |
| Objectives | 4 | Considering that these controversial results, we performed this meta-analysis to derive a more precise, comprehensive assessment for the overall diagnostic value of CTA and MRA in evaluation of vascular access in hemodialysis patients. To the best of our knowledge, this is the first meta-analysis on CTA and MRA in evaluation of vascular access in hemodialysis patients. | Introduction |
| **METHODS** | | |  |
| Protocol and registration | 5 | No |  |
| Eligibility criteria | 6 | (1) evaluation of the diagnostic performance of CTA or MRA for detecting or evaluating stenosis, (2) On per-segment or per-patient statistical basis, presentation of information for true-positive (TP), false-positive (FP), true-negative(TN) and false-negative(FN) results either found or calculated from data in the original published study, (3) Articles were published in English, (4) DSA or surgery should be the reference standards. | Inclusion and Exclusion Criteria |
| Information sources | 7 | Pubmed, MEDLINE, EMBASE, Cochrane Library database were all searched (Last search was updated on May, 2013). | Publication search |
| Search | 8 | The following terms were used in searching: (vascular access or arteriovenous fistula or arteriovenous graft) and (hemodialysis or uremic or renal failure or renal disease or kidney failure or kidney disease) and (computed tomography angiography or magnetic resonance angiography or CT angiography or MR angiography or CTA or MRA). | Publication search |
| Study selection | 9 | Eligibility assessment was performed independently in an unblinded standardized manner by 2 reviewers. Disagreements between reviewers were resolved by consensus. | Data Extraction and Quality Assessment |
| Data collection process | 10 | Data extraction was performed independently and discrepancies were resolved by discussion by 2 reviewers. | Data Extraction and Quality Assessment |
| Data items | 11 | Information was extracted from each included studies: characteristics of the study population, methodological details for CTA, MRA, reference standard and outcome data (TP, FP, TN, FN). | Data Extraction and Quality Assessment |
| Risk of bias in individual studies | 12 | To ascertain the validity of eligible studies, pairs of reviewers working independently. There is blinding of patients, health care providers, data collectors, and outcome assessors in individual studies. | Data Extraction and Quality Assessment |
| Summary measures | 13 | The meta-analyses were performed by computing area under SROC and Diagnostic Odds ratios (DORs) using random-effects or fixed model to assess the strength of association. DOR and 95% confidence intervals for each diagnostic performance were calculated. Quantitative analyses were performed on heterogeneity and publication bias. | Meta Analysis |
| Synthesis of results | 14 | We tested for heterogeneity with the the Chi square-test based Cochrane Q-test to measure inconsistency (the percentage of total variation across studies due to heterogeneity) of effects. The advantages of this measure of inconsistency (termed I2) are that it does not inherently depend on the number of studies and is accompanied by an uncertainty interval. In each study included, the response data available were the number of true positive, false positive, false negative, false positive. | Meta Analysis |

Page 1 of 2

| **Section/topic** | **#** | **Checklist item** | **Reported on page #** |
| --- | --- | --- | --- |
| Risk of bias across studies | 15 | For each study we plotted the effect by the inverse of its standard error. The symmetry of such ‘funnel plots’ was assessed both visually and formally with Begg’s test and Egger’s test, to see if the effect decreased with increasing sample size. | Meta Analysis |
| Additional analyses | 16 | One of main causes of heterogeneity is threshold effect in test accuracy studies. The threshold effect arises owing to different thresholds or cut-offs used in different studies to define a positive (or negative) test result. | Meta Analysis |
| **RESULTS** | | |  |
| Study selection | 17 | Our search strategy identified 333 primary studies. After ruling out the obviously irrelevant abstracts, 26 studies were left and their full texts were obtained. The search initially yielded 333 primary studies. Finally there were 16 studies in 15 articles included in the meta-analysis | Eligible studies |
| Study characteristics | 18 | Characteristics of included studies: For each technique, the weighted summary of sensitivity, specificity, positive likelihood ratio, negative likelihood ratio,  Methods  All 16 studies finally selected for the review were published in English. Using CTA or MRA to detect or evaluate hemodialysis access stenosis as index test and DSA or surgery as standard test (golden standard).  Participants  500 patients with native hemodialysis access who underwent CTA/MRA and DSA/surgery. | Eligible studies |
| Risk of bias within studies | 19 | Not depicted. |  |
| Results of individual studies | 20 | | Author | Year | Patient Num | Access Type | Noninvasive Modalities | TP | FP | FN | TN | | --- | --- | --- | --- | --- | --- | --- | --- | --- | | Cansu[10] | 2013 | 41 | AVF and AVG | CTA | 34 | 2 | 1 | 30 | | Wasinrat[24] | 2011 | 21 | AVF | CTA | 32 | 6 | 0 | 109 | | Rooijens[21] | 2008 | 15 | AVF and AVG | CTA | 9 | 1 | 2 | 124 | | Heye[16] | 2009 | 36 | AVF | CTA | 46 | 8 | 5 | 103 | | Dimopoulou[12] | 2011 | 24 | AVF and AVG | CTA | 37 | 0 | 2 | 33 | | Ko[17] | 2005 | 36 | AVF and AVG | CTA | 126 | 2 | 2 | 69 | | Lin[19] | 1998 | 9 | AVF | CTA | 6 | 0 | 0 | 3 | | Cavagna[11] | 2000 | 13 | AVF | CTA | 11 | 0 | 0 | 2 | | Froger[15] | 2005 | 48 | AVF and AVG | MRA | 68 | 3 | 2 | 209 | | Waldman[23] | 1996 | 13 | AVF and AVG | MRA | 8 | 1 | 0 | 33 | | Takahashi[22] | 2004 | 15 | AVF | MRA | 16 | 3 | 3 | 19 | | Duijm[14] | 2006 | 101 | AVF and AVG | MRA | 18 | 1 | 0 | 82 | | Doelman[13] | 2005 | 81 | AVF and AVG | MRA | 106 | 7 | 5 | 315 | | Planken[20] | 2003 | 15 | AVF and AVG | MRA | 10 | 4 | 0 | 1 | | Cavagna[11] | 2000 | 13 | AVF | MRA | 10 | 0 | 1 | 2 | | Laissy[18] | 1999 | 19 | AVF and AVG | MRA | 11 | 1 | 1 | 6 | | Table1 |
| Synthesis of results | 21 | Overall AUC of CTA and MRA was 0.988 and 0.982, which suggesting good diagnostic accuracy. Pair-wise comparisons confirmed no statistical difference between CT and MR imaging performance.   | Modality |  | Sensitivity | Specificity | PLR | NLR | DOR |  | | --- | --- | --- | --- | --- | --- | --- | --- | | CTA |  |  |  |  |  |  |  | |  | Pooled estimates | 0.962 | 0.961 | 17.64 | 0.06 | 393.69 |  | |  | 95%CI | 0.93-0.98 | 0.94-0.98 | 11.17-27.84 | 0.03-0.12 | 155.20-998.67 | | |  | P value* | P=0.068 | P=0.119 | P=0.369 | P=0.179 | P=0.287 |  | |  | I2 value | 46.90% | 39.00% | 7.90% | 31.20% | 18% |  | | MRA |  |  |  |  |  |  |  | |  | Pooled estimates | 0.954 | 0.971 | 13.36 | 0.075 | 211.47 |  | |  | 95%CI | 0.920-0.976 | 0.955-0.982 | 2.42-73.95 | 0.039-0.144 | 46.36-964.67 | | |  | P value* | P=0.327 | P=0.000 | P=0.000 | P=0.203 | P=0.001 |  | |  | I2 value | 13.20% | 79.30% | 95.20% | 28.20% | 70.40% |  | | Data synthesis,  Table2 |
| Risk of bias across studies | 22 | The results of Deeks’ funnel plot asymmetry test (P =0.035) showed strong evidence for publication bias for MRA studies but no publication bias was indicated for CTA studies. | Data synthesis |
| Additional analysis | 23 | Threshold effect analyze | Threshold effect analyze |
| **DISCUSSION** | | |  |
| Summary of evidence | 24 | Overall, the evidence is sufficiently strong to determine the diagnostic performance of CTA and MRA | Discussion |
| Limitations | 25 | Outcome level: we could not present the exact reasons for heterogeneity which was observed for pooled specificity, PLR, DOR of MRA.  Study and review level: there are still many variables which differed among studies regarding patient position during examination, patient characteristics, acquisition protocol, image analysis technique, indication for imaging, interobserver variability, and quality of studies, especially patient position. Bur these factors were not taken into account and the effect of these variables could not be examined because of variation in data presentation or incomplete reporting of data. | Discussion |
| Conclusions | 26 | Implications for research  CTA and MRA had similar, excellent accuracy for detecting stenosis of hemodialysis vascular access. There is no statistical difference between the diagnostic performance of CTA and MRA. Both techniques may function as an alternative or an important complement to conventional digital subtraction angiography and may be able to help guide medical management. | Conclusions |
| FUNDING | | |  |
| Funding | 27 | This study was supported by grant no. 81171333 and no. 81230030 from the National Natural Science Foundation of China | online submission system |

*From:*  Moher D, Liberati A, Tetzlaff J, Altman DG, The PRISMA Group (2009). Preferred Reporting Items for Systematic Reviews and Meta-Analyses: The PRISMA Statement. PLoS Med 6(6): e1000097. doi:10.1371/journal.pmed1000097

For more information, visit: **www.prisma-statement.org**.

Page 2 of 2
